# Embryonic Exposure to Domoic Acid Increases the Susceptibility of Zebrafish Larvae to the Chemical Convulsant Pentylenetetrazole

**DOI:** 10.1289/ehp.10344

**Published:** 2007-08-02

**Authors:** Jessica A. Tiedeken, John S. Ramsdell

**Affiliations:** Marine Biotoxins Program, Center for Coastal Environmental Health and Biomolecular Research, National Oceanic and Atmospheric Administration, National Ocean Service, Charleston, South Carolina, USA

**Keywords:** *Danio rerio*, domoic acid, EthoVision, pentylenetetrazole, seizures, zebrafish

## Abstract

**Background:**

Domoic acid (DA) is a neurotoxin produced by diatoms of the genus *Pseudo-nitzschia* that targets the limbic system to induce tonic–clonic seizures and memory impairment. *In utero* DA exposure of mice leads to a reduction in seizure threshold to subsequent DA exposures in mid-postnatal life, and similar studies have shown neurotoxic effects in rats that were delayed until adolescence.

**Objective:**

We used *in ovo* microinjection of zebrafish (*Danio rerio*) to characterize the effect of embryonic exposure of DA on seizure-inducing agents later in life as an alternative species model to screen environmental contaminants that might induce a fetal-originating adult disease.

**Methods:**

Embryos were microinjected within hours of fertilization to DA concentrations ranging from 0.12 to 1.26 ng/mg egg weight. Seven days later, the larval animals were characterized for sensitivity to the chemical convulsant pentylenetetrazole (PTZ), an agent that is well-defined in laboratory rodents and, more recently, in zebrafish.

**Results:**

*In ovo* DA exposure, most significantly at 0.4 ng/mg, reduces the latency time until first PTZ seizure in larval fish and increases the severity of seizures as determined by seizure stage and movement parameters. The interaction between *in ovo* DA exposure and PTZ caused seizure behaviors to individually asymptomatic doses of PTZ (1.0 and 1.25 mM) and DA (0.13 and 0.22 ng/mg).

**Conclusion:**

These studies demonstrate that *in ovo* exposure to DA reduces the threshold to chemically induced seizures in larval fish and increases the severity of seizure behavior in a manner that is consistent with *in utero* studies of laboratory rodents.

Produced by multiple diatom species across several genera, most notably *Pseudo-nitzschia,* the toxin domoic acid (DA) is responsible for human poisonings (Wright et al. 1989), which are clinically diagnosed as amnesic shellfish poisoning (ASP) (Perl et al. 1990). Planktivorous fish are primary vectors for DA in marine food webs, exposing several marine birds and mammals to this neurotoxin (Lefebvre et al. 1999; Scholin et al. 2000; Work et al. 1993). DA is structurally related to kainic acid, a subgroup of the neurotransmitter glutamate family, and therefore activates ionotropic AMPA and kainate subtypes of glutamate receptors (Slevin et al. 1983). Receptor activation leads to glutamate release and coincident glutamate-NMDA (*N*-methyl-d-aspartate) receptor–mediated induction of immediate response genes (Peng et al. 1994; Ryan et al. 2005) and excito-toxicity (Novelli et al. 1992).

Consistent with symptoms observed in humans (Teitelbaum et al. 1990), similar indicators of DA exposure have been repeatedly documented in mice, rats, and monkeys (Tasker et al. 1991; Tryphonas et al. 1990a, 1990b). Of particular note are the behavioral manifestations of seizures, including stereotypic scratching, tremors, and tonic–clonic seizures, which have been used to quantitate toxicity scores (Tasker et al. 1991). In addition to behavioral symptoms, DA exposure has been reported to cause neuroexcitation and extensive degeneration in brain tissues (Peng et al. 1994; Scallet et al. 1993), along with learning and memory deficiency in humans and experimental animals (Clayton et al. 1999; Petrie et al. 1992; Sutherland et al. 1990; Teitelbaum et al. 1990). The pyramidal cells in the amnion horn region of the hippocampus exhibit the most damage, with DA also prominently affecting the septum and olfactory bulb (Colman et al. 2005; Scallet et al. 1993). The pathways through the hippocampus, which are responsible for learning and spatial memory, are highly prone to seizures (Ramsdell 2007). Thus, the susceptibility for seizures in areas of the brain responsible for learning and memory assimilation allows for the overlap in investigative models regarding the role of environmental toxicants on epilepsy, as well as on memory and learning impairments.

Subsequent to DA toxicity characterization in adult animals, substantial research has examined developmental toxicity, in large part to better understand through laboratory studies the high levels of DA toxicity observed in pregnant California sea lions (Brodie et al. 2006; Gulland et al. 2002). Susceptibility to DA has been characterized both in prenatal and postnatal rats and mice. Postnatal rats are highly sensitive to DA, responding with seizure behavior to doses 40 times lower per body weight than adults (Xi et al. 1997). As the animals mature through postnatal life, the outward manifestation of seizure behavior changes, which is likely a reflection of growth surge associated with migration of new neuronal pathways and synaptogenesis (Doucette et al. 2000, 2004). Concurrently, the maturation of renal function correlates with the decrease in susceptibility of neonates to DA as they age. In addition, although an effective dose of DA is present in milk from exposed lactating rats, parental transfer of toxin by lactation remains well below symptomatic doses due to low oral adsorption (Maucher and Ramsdell 2005).

Prenatal rats are susceptible via *in utero* exposure to DA, with symptoms usually showing up later during postnatal life. *In utero* exposure of gestational day (GD) 13 mice to DA decreases the threshold to DA-induced seizures by postnatal day (PD) 10 (Dakshinamurti et al. 1993). In rats it leads to a reversal in the normal sex-based difference in learning and decreases the threshold to scopolamine-induced memory impairment as juveniles (Levin et al. 2005). These later effects appear specific to the prenatal exposure, as rats treated at PD0–PD1 did not show these deficits as juveniles (Levin et al. 2006). Neurogenesis in mice and rats occurs during the second half of gestation (GD12–GD20), as the brain growth spurt and synaptogenesis is postnatal (Dobbing and Sands 1979). This indicates that neurogenesis represents the sensitive window of exposure to DA during development. Research characterizing the enhanced sensitivity to convulsant and amnestic drugs expressed later in life as result of *in utero* exposure is part of an emerging discipline known as the fetal basis to adult disease, which predicts a peak impact in the earlier *in utero* development for increased susceptibility to dysfunction later in life (Heindel 2005).

Zebrafish are increasingly being used as models for neurotoxicity, and the accessibility of the embryos to manipulation and observation are ideal for developmental studies (Ton et al. 2006). DA symptomatology in early development has been investigated in zebrafish embryos and larvae, and has also been found to emulate symptoms characteristic of rodents. When eggs are injected with DA, within hours of fertilization and before the onset of the first phase of neurogenesis, embryos exhibit marked tonic–clonic type convulsions at 2 days postfertilization (dpf) and constant rapid pectoral fin motion beginning at 5 dpf (Tiedeken et al. 2005). Distinct seizure patterns have also been characterized in 7-dpf larval zebrafish exposed by bath to the seizure-inducing agent pentylenetetrazole (PTZ) (Baraban et al. 2005). PTZ is a competitive antagonist of the GABA_A_ (gamma-aminobutyric acid type A) receptor, binding to the picrotoxin site of the channel and acting via an allosteric interaction on the channel (Huang et al. 2001; Ramanjaneyulu and Ticku 1984; Rehavi et al. 1982). Its action on the GABA_A_ receptor causes blockage of chloride conductance and formation of inhibitory postsynaptic potentials, which increases glutamatergic excitability (Macdonald and Barker 1978). In larval zebrafish, three stages of seizure activity have been characterized and validated using electrophysiologic and pharmacologic analysis. The first stage is defined as a general overall increase in activity, followed by rapid whirlpool-like swimming in the second stage, and concluding with full body convulsions in stage three (Baraban et al. 2005).

In this study we used zebrafish embryos as an alternate species to quantify the persistent effects of early-life exposure to DA. By using PTZ as an acute chemical convulsant, well-defined seizure behaviors have been analyzed in larval fish exposed to DA prior to neurogenesis. The correspondence of this study to parallel studies in rodents indicates that results in microinjected zebrafish embryos can be more widely applied to screen for environmental contaminants that may induce a fetal-originating adult disease.

## Materials & Methods

### Zebrafish

Thirty male and female zebrafish (*Danio rerio*) of AB wild-type strain were obtained from Zebrafish International Resource Center (ZIRC; Eugene, OR) and allowed to breed at random for embryo production. Fish were kept on a 14 hr light:10 hr dark cycle in a recirculating aquarium rack system (Aquatic Habitats, Apopka, FL); water conditioning and environmental quality was maintained according to the manufacturer’s instructions and *The Zebrafish Book* (Westerfield 2000). Utmost care was used to insure that the animals were treated humanely, and in cases where distress could not be alleviated, the animals were euthanized. Zebrafish were fed twice daily with Zeigler Adult Zebrafish Diet (Aquatic Habitats) and on afternoons before breeding the diet was supplemented with live *Artemia*. Breeding inserts were placed in tanks with a plastic plant to collect fertilized eggs (embryos) in the morning. The embryos were removed from the bottom of the tank within the first hour of light, and washed with sterile water.

### *DA *in ovo *microinjection*

DA, along with all other reagents, was purchased from Sigma Chemical (St. Louis, MO). The DA was resuspended in sterile phosphate-buffered saline (PBS) to a stock concentration of 10 mg/mL. Concentrations were diluted in PBS in preparation for a 2.4-nL injection to create 0.126, 0.22, 0.40, 0.71, and 1.26 ng DA per embryo (1.4 mg wet weight). Concentrations were determined on the basis of previous work by Tiedeken et al. (2005).

Healthy embryos between 1K-cell and high-oblong cell stages, which occurs around 3–4 hr postfertilization (hpf) depending on temperature conditions (Kimmel et al. 1995), were aligned in troughs imbedded in an agarose plate as described in *The Zebrafish Book* (Westerfield 2000). Each plate, one for controls and one for DA injections, was doused with 12.5% Hanks solution (Westerfield 2000) to submerge embryos. All injection procedures were observed using a Leica MZ 12 stereomicroscope (Leica Microsystems Inc., Bannockburn, IL). A pulled (P-87; Sutter Instrument Co., Navato, CA) and beveled (BV-10; Sutter Instrument Co.) alumino-silicate filament micropipette (o.d. 1 mm; Sutter Instrument Co.) was filled with a known DA concentration using a microloader pipette tip (Eppendorf North America, Westbury, NY) and placed in a micro-manipulator (MO-150; Narashige Group, Long Island, NY). A nitrogen gas pico-injector (PLI-100; Harvard Apparatus, Holliston, MA) was calibrated to consistently produce 2.4 nL of injection material.

After injection, embryos were removed from the plates and transferred to petri dishes (Corning Life Sciences, Acton, MA) with E3 media (Brand et al. 2002), which was replaced daily following removal of nonviable embryos. Embryos hatched around 3 dpf and the larvae were raised to 7 dpf in a 28°C incubator, with frequent observational monitoring for DA toxicity. On the sixth day, media was slowly changed to Ringers solution (Westerfield 2000) to allow for the embryos to acclimate before trials.

### PTZ challenges

For each DA dose, including vehicle-injected and noninjected, 7-day-old larvae were transferred to individual wells of 96-well plates (Costar 3610; Corning Life Sciences) with 50 μL fresh Ringers solution and allowed to acclimate for a few hours. Plates were arranged so that at least 10 larvae were available for each PTZ dose. Noticeable DA symptomology, pectoral fin movements, and deformities were noted at this time. The plate was placed on the white light box at the bottom of a Tracksys Tower Filming system (Tracksys Ltd., Nottingham, UK), and a baffle was lowered to reduce ambient light. The tower was connected to a computer running EthoVision Pro 3.1 behavioral tracking software (Noldus Information Technology Inc., Leesburg, VA). A 3-min baseline trial was tracked using EthoVision and simultaneously recorded using an MPEG encoder (MPEG Pro EMR100; Canopus, San Jose, CA). The medium was replaced with one of eight concentrations of PTZ (0, 1, 1.25, 2.5, 5, 10, 15, or 25 mM) dissolved in Ringers solution. The response was recorded for a total of 20 min with an additional 3-min EthoVision trial occurring after 15 min of exposure had lapsed. After observations, all subjects were euthanized with a lethal concentration of MS-222 Tricaine (ethyl-3-aminobenzoate methane-sulfonate salt; Sigma Chemical).

### Analysis

DA-induced morphologic and behavioral differences were compared using recorded images from a Sony RGB camera (DXC-390; Sony Corporation, Tokyo, Japan) attached to the Leica stereomicroscope. Recordings of the larvae after the addition of PTZ were reviewed for behavioral changes and seizures. Time to reach the first definitive stage II seizure (seizure latency) was also noted. Because the viability of the larvae varied due to the dose of DA, sample size varied from 12 to 40. We obtained the distance moved and the mobility parameters from EthoVision tracking program analysis, and baseline parameters were subtracted out before calculation of statistics. Because of the time-dependent nature of the trials, those individuals who did not track properly were excluded; however, we used a minimum of 10 individuals per DA/PTZ combination in all calculations. Two-way analysis of variance (ANOVA) followed by Bonferroni means comparison test (Prism version 4; GraphPad Software Inc., San Diego, CA) were used to analyze the compounding effects of both DA and PTZ responses on the larval fish.

## Results

### Acute embryonic response to DA

We assessed the acute response to DA microinjection for the embryonic period, concluding with hatching around 3 dpf and into the larval period. Embryos exposed *in ovo* with low doses of 0.126 and 0.22 ng/mg DA displayed no visual symptomatic behavioral responses to the toxin. Two days after injection (2 dpf), members of the 0.4-, 0.71-, and 1.26-ng/mg groups exhibited DA-induced convulsive behavior that endured for 12–24 hr. As described previously, the behavior resembled tonic–clonic convulsions manifested as a whole-body contraction with a shuddering motion (Tiedeken et al. 2005). Each contraction lasted approximately 3–5 sec, and the percentage convulsing increased with dose. In addition, the higher dose groups had individuals with spinal deformities and incessant pectoral fin motions which were observed as early as 4 dpf and persisted through the conclusion of the experiment at 7 dpf. Many larval individuals in the two highest DA cohorts, especially those suffering from the spinal deformities, exhibited an absence of touch response and an overall inability to move. The percentage of symptomatic individuals and severity of DA toxicity increased proportionately with dose. In all analyses, the vehicle-injected controls and the noninjected controls exhibited asymptomatic behavior, and minor differences arising in PTZ trials were determined to be insignificant (*p* > 0.05).

### Sustained larval response to DA

#### PTZ seizure characterization

**Figure 1:**
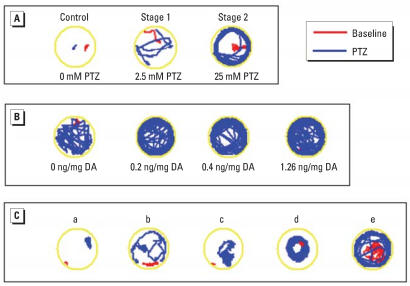
EthoVision tracks (3-min duration) recorded before (baseline) and 15 min after PTZ addition. Lines show the track of fish movement. (*A*) Control (0.0 ng/mg DA) larvae exposed to 0, 2.5, and 25 mM PTZ, exhibiting seizure behaviors; no control larvae exhibited Stage III convulsions only. (*B*) Larvae demonstrating increased seizure activity across increasing DA concentrations when exposed to 5 mM PTZ. (*C*) Track examples of differences in PTZ (25 mM) response resulting from embryonic exposure to various prior DA doses: a) 1.26 ng/mg DA, stage III convulsions only; b) 1.26 ng/mg DA, increased movement originating from increased pectoral fin response only; c) 0.71 ng/mg DA, increased convulsive movement; d) 1.26 ng/mg DA, circular motion resulting from spinal deformities; and e) 0.4 ng/mg DA, increased movement in a similar pattern to, but without completing, a defined stage II seizure.

**Figure 2:**
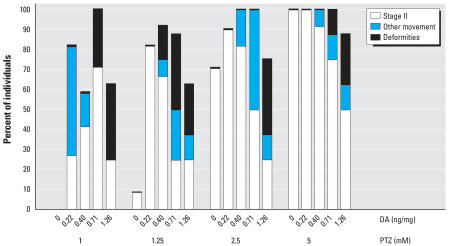
Percentage of individuals across DA doses exhibiting a behavioral response to exposure to PTZ within 20 min observation. Fish that exhibited a writhing motion or convulsive-only motion without a definable whirlpool action were grouped as other movements. “Deformities” include the percentage of fish whose physical deformities from DA prohibited them from exhibiting stage II seizures. The number of 0.13-ng/mg DA-exposed larvae responding is comparable to 0 ng/mg and has been omitted. PTZ doses > 5 mM exhibited 100% response across all DA doses (data not shown).

A stage II seizure to PTZ in zebrafish consists of an easily identifiable, rapid, whirlpool swimming motion (Figure 1A), which is clearly distinguishable from the first stage of generalized motion (Baraban et al. 2005). The 7-dpf larvae with prior DA exposure all showed an increased seizure response corresponding to the concentration of PTZ, ranging from 1 to 25 mM in bathing solution. Embryos exposed to a midrange effective dose of 5 mM PTZ responded with increased severity to the seizure inducing agent as *in ovo* DA dose increased (Figure 1B). Larvae that were immobilized (from the 1.26 ng/mg DA cohort) responded to the PTZ with whole-body (head touches tail) contractions, severe tremors, and/or an increase of pectoral fin motion allowing for slow spinning movements (Figure 1C). Across all DA and PTZ combinations, individuals showed a writhing motion in addition to defined seizure stages. This behavior was not observed in larvae without *in ovo* DA exposure, and no seizure activity was noted in the absence of PTZ (Ringer’s solution), regardless of DA exposure. These writhing and spinning movements exhibited at the higher *in ovo* DA doses (≥0.4 ng/mg) impaired some of the embryos from reaching a discernable stage II seizure behavior, and many went directly into stage III (convulsive) activity. At lower doses of PTZ (< 5 mM), increasingly more larvae were exhibiting a stage II response to PTZ within the observation time (Figure 2). At concentrations of PTZ > 5 mM, all embryos exhibited a response, regardless if it was a recognizable stage II seizure.

#### Analysis of seizure latency

**Figure 3:**
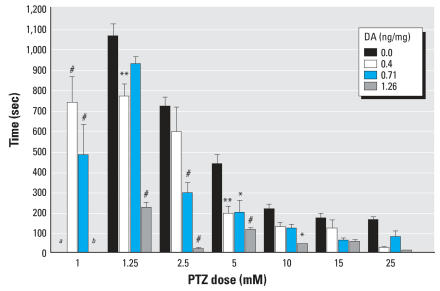
Amount of time (mean ± SE) for a definable stage II seizure after immersion in PTZ media. ^***a***^No fish had a stage II response at this dose. ^***b***^All fish in this group showed a motion response but were unable to complete a definitive stage II seizure. *p < 0.05, **p < 0.01, and ^#^*p* < 0.001 compared with control.

The time required between PTZ exposure and initiation of the first stage II seizures in the larvae was measured for seizure latency. In control fish, the seizure latency was approximately 800 sec for 2.5 mM PTZ to about 150 sec for 25 mM PTZ. All larvae exposed *in ovo* to DA showed a time reduction in stage II seizure latency, and at high PTZ concentrations (> 10 mM) a stage II seizure was observed within the first 60 sec (Figure 3). Commencing with the 0.22-ng/mg group, DA-treated embryos reached a stage II seizure activity at the lowest PTZ dose (1 mM), a dose that elicited no seizure response in the control groups. The *in ovo* 1.26 ng/mg DA exposure group also exhibited a behavioral response to 1 mM PTZ within 100 sec of exposure, although it was indefinable as a stage II seizure. Significant time reductions of seizure latencies were also noted across other dose combinations (Figure 3). Even the lowest DA treatment (0.13 ng/mg) showed a significant reduction in time (*p* < 0.05) at 1.25 and 2.5 mM PTZ concentrations (data not shown). Although not included in this analysis, those *in ovo* exposed embryos that did not have a defined stage II reaction still showed a unique motion response to PTZ within the first 200 sec. This may contribute to the insignificant decrease from *in ovo* DA exposure at PTZ levels of ≥10 mM because a larger percentage went straight into a stage III seizure or did not exhibit a distinguishable seizure.

#### Analysis of distance moved

**Figure 4:**
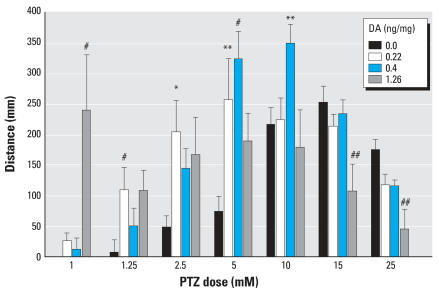
Increase in total distance traveled above baseline (mean ± SE) after a 15-min bath exposure to PTZ across DA doses. Values significantly greater than vehicle: *p < 0.05, **p < 0.01, and ^#^*p* < 0.001. Values significantly lower than vehicle: ^##^*p* < 0.05.

Stage I, defined as a generalized increase in activity, and stage II seizures both exhibit increased movement around the well, whereas stage III seizures (stationary convulsions) exhibit a reduction in movement. When tracked with EthoVision after 15-min immersion in various concentrations of PTZ, DA-exposed cohorts (0.22 and 0.4 ng/mg) showed a significant increase (*p* < 0.01) in distance moved. At bath concentrations of PTZ up to 5 mM, larvae that had been exposed *in ovo* to doses as low as 0.22 ng/mg DA traveled at least twice the distance as vehicle-microinjected embryos (Figure 4). As the larvae exposed to higher *in ovo* doses of DA progressed through the stages of seizures faster, they reached a stage III seizure sooner than the controls. EthoVision responded by tracking less distance moved at these levels. High DA doses showed significant (*p* < 0.05) decreases in distance moved when exposed to 15 and 25 mM PTZ. Members of the 1.26-ng/mg DA cohort traveled 5 times the distance of any other group when exposed to 1 mM PTZ, despite never exhibiting a definable stage II seizure.

#### Analysis of larval mobility

**Figure 5:**
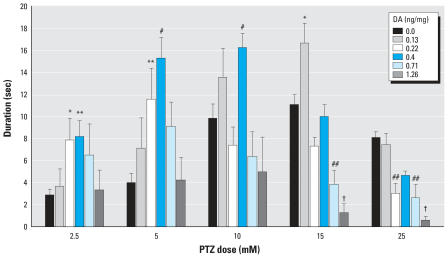
Duration of time spent in a strongly mobile state (mean ± SE) after exposure to PTZ. Values significantly greater than vehicle: *p < 0.05, **p < 0.01, and ^#^*p* < 0.001. Values significantly lower than vehicle: ^##^*p* < 0.05, and ^†^*p* < 0.01.

The mobility parameter of the EthoVision software is able to detect distance traveled along with stationary movement of the individual. Stage III seizures, a convulsive behavior, show no distance movement but do exhibit stationary body movement. Embryos treated with DA showed a significant increase in the duration of time spent in a strongly mobile state, a measurement that occurred mostly at stage II (Figure 5). Members of the lowest dose cohort of DA (0.13 mg/kg) showed a significant increase (*p* < 0.05) in mobility duration at 15 mM PTZ; this parameter incorporates not only stage II movement but stage III. Other doses of PTZ for this 0.13-ng/mg DA cohort did not clear a statistical cutoff (*p* < 0.05) for increased mobility but did show a significant decrease in duration of immobility (*p* < 0.01), indicating that the fish had increased overall activity (data not shown) at 1.25, 2.5, and 5 mM PTZ. Larvae that were exposed *in ovo* to 0.4 ng/mg DA showed the greatest increase in mobility with exposure to 5 and 10 mM PTZ. The fish in this cohort also spent significantly (*p* < 0.001) more time in a strongly mobile state at 5 and 10 mM PTZ as well as 2.5 mM PTZ (*p* < 0.01) (Figure 5). Although the distance moved did not significantly increase (*p* > 0.05) compared with the vehicle-exposed group, the members of the highest DA group (1.26 ng/mg) showed a significant (*p* < 0.001) decrease in duration of strong mobility and an increase in mobility to higher PTZ doses, thereby indicating that these larvae were undergoing convulsive motions. Because of the large variability between individuals, greater statistical differences may come to fruition with additional trials.

## Discussion

The present study demonstrates that *in ovo* exposure to DA increases the susceptibility of larval zebrafish to the seizure-inducing agent PTZ. Furthermore, effects of early-life DA exposure were observed after exposure to non-symptomatic doses of PTZ, demonstrating that DA toxicity can be unnoticeable until provoked by a heterologous agent. Larval fish exposed to DA as embryos also showed a significantly greater seizure response to symptomatic doses of PTZ. This was clearly demonstrated through increased movements and behavioral responses. DA exposure allowed for an increased response, both in number of animals responding and in time to reach a response within the observational window of 20 min. Although only stage II whirlpool motion activity was scored, there was an overall change of behavior noted in the DA-exposed groups. Many larvae, especially at higher DA doses, lacked touch response and movement; however, exposure to PTZ in these animals led to an immediate motion response. These responses ranged from simple increased movement (stage I) to immediate stage III seizures without advancement through the typical progression of seizure behavioral stages. Overall, these responses provide evidence that DA causes an early alteration in the potential excitability of seizure-prone neuronal pathways in larval zebrafish.

Although the time frame for neurodevelopment in zebrafish is rapid compared with mammalian models, the delineation of major brain regions and their developmental plan is well conserved. In zebrafish, the brain is apparent toward the end of the gastrula period (9 hpf), which is > 5 hr after DA was microinjected into the yolk sac. By the mid-pharyangula period (28 hpf), the major forebrain regions are in place, including the diencephalon (comprised of dorsal thalamus and ventral hypothalamus) and the telencephalon (comprised of the pallium and lateral division) (Mueller and Wullimann 2003). These areas have shown correspondence to the mammalian hippocampus and subpallium (Rodriguez et al. 2002). Although these regions are conserved, the anamniotic zebrafish—unlike amniotic mammals—undergo two distinct processes of neurodevelopment that are needed to accommodate the early developing larval life form. This results in an early phase of neurogenesis that occurs within the first day (16 hpf) to form the simple larval brain plan, and a second wave following hatching, commencing around 48 hpf. This second wave of neurogenesis, via migration of neurons from the ventricular surface of the larval brain, will override much of the circuitry created in embryonic development (Mueller and Wullimann 2003). Although all the elements of neuronal development are present in the zebrafish model, the difference in processes may vary the comparison to rodent models.

DA administered *in ovo* induces acute neurotoxic effects as the brain matures and gains responsiveness to the toxin. Effects of DA do not become apparent until tonic–clonic convulsions begin at the end of the pharyangela phase (2 dpf) (Tiedeken et al. 2005). This indicates that the early neural network of the larval brain is responsive to this glutamatergic convulsant. Interestingly, this effect is transient and lasts only until onset of the hatching phase, about 6 hr later. However, other neurologic effects appear after hatching, including stereotypic-like pectoral fin flapping beginning at 5 dpf and, at higher doses, a loss of touch response. The 7-dpf larvae, which have completed the final absorption of the yolk sac, have been well characterized for seizure response to PTZ by behavioral, electrographic, and pharmacologic criteria and were determined to develop multiple phases of seizure activity mediated by glutamatergic:GABAergic neurotransmitter systems (Baraban et al. 2005). The PTZ larval symptomology is comparable with symptoms observed in postnatal and adult rodents exposed to the convulsive agent.

We have extended our acute studies of *in ovo* DA exposure in zebrafish to examine the persistent effects of DA later in life by examining the response to the chemical convulsant PTZ. PTZ is very well characterized as a convulsant and is a well-established acute seizure model in rats and mice to investigate pharmaceuticals that may control seizure susceptibility (White et al. 1995). The studies presented here show that zebrafish demonstrate similar persistent susceptibility to excitotoxins, as has been determined in rodents. When pregnant mice are exposed to an asymptomatic dose of DA on GD13, the postnatal offspring show no evidence of spontaneous seizures, yet demonstrate increasing irregularities in electroencephalogram recordings in postnatal life (Dakshinamurti et al. 1993). The postnatal mice, however, did show a reduced threshold to DA-induced seizures compared with mice that had no prior *in utero* exposure. Like the larval zebrafish exposed to DA *in ovo*, the neonatal mice exposed to DA *in utero* exhibited a reduction in seizure latency and greater seizure activity (Dakshinamurti et al. 1993). The effects of *in utero* DA exposure in mice were not evident until postnatal life; however, acute neurotoxic effects in neonates cannot be discounted. By contrast, acute effects of *in ovo* DA exposure in zebrafish are readily apparent before hatching because of easily visualized embryogenesis, allowing for identification of doses causing transient effects during development that may not be readily apparent in the mouse model. Observation of early seizure behavior *in ovo* allows for establishment of threshold DA dose values relative to neurologic development in the embryo.

In the mouse model, effects were analyzed through neuronal histology, which showed no evident altered morphology of the hippocampus on the first postnatal day, yet began to appear midway through the postnatal period (PD14) (Dakshinamurti et al. 1993). This time corresponds to the postnatal growth surge in rodents, which includes apoptosis, migration, and synaptogenesis of the brain tissue. The neuronal damage in the mouse hippocampus was associated with increased chemical excitability, as indicated by an increased ratio of glutamate:GABA in the brain and receptor density determined by increased kainic acid binding sites (Dakshinamurti et al. 1993). The increased sensitivity to PTZ seizures in zebrafish is consistent with an action of DA to increase the chemical excitability of the balance between glutamate and GABA neurotransmitters. Neurogenesis in the mouse hippocampus begins at GD13, and *in utero* exposure to DA at this time suggests that alteration in neurogenesis may ultimately lead to organization, which promotes excitatory (glutamatergic) overinhibitory (GABAerigic) balance in the hippocampus. Alteration of neurogenesis by *in utero* exposure of mice with mitotic inhibitors also leads to increased vulnerability to seizures in postnatal life, suggesting that alteration in expansion of clusters of neuroprogenitor cells can alter the excitability of the developed brain (Paredes et al. 2006). In the present study the *in ovo* exposure of DA precedes both waves of anamniotic neurogenesis, indicating targeted mechanisms for DA toxicity in rodents may also be activated in the zebrafish.

The *in utero* effects of DA are not limited to seizure response but also extend to memory and learning behaviors. Persistent effects on learning and memory have been examined in rats treated with DA *in utero* on GD13 (Levin et al. 2005). DA led to a normalization of sex-based differences that occur in radial-arm maze testing. Additionally, when the rats were exposed to the amnestic drug scopolamine, animals injected with DA *in utero* showed significant impairment in response time. These results indicate that prenatal exposure to DA in rats gives rise to a persistent effect of decreased cognitive reserves. By contrast, rats exposed to DA on PD0 and tested as juveniles and adults did not show the above deficits in learning and cognitive reserve, but lesser effects were manifested in hypoactivity (Levin et al. 2006). Given that the peak in neurogenesis is complete by birth in rats, these data suggest that this window represents the most susceptible time frame for DA toxicity.

It is possible that *in ovo* exposure to DA in zebrafish may also provide a means to screen for fetal-originating deficits in special memory and cognitive reserve. DA effects on seizure activity and memory appear to share common pathways gated by the dentate granule cells of the hippocampus (Ramsdell 2007). Development of the region structurally corresponding to the hippocampus (lateral pallium) in the zebrafish differs morphologically but still appears to retain the functionality for spatial memory (Rodriguez et al. 2002). Indeed, adult zebrafish have been used in memory studies and increasingly more behavioral studies with specially designed mazes and behavioral monitoring software (Levin and Chen 2004). Adult zebrafish are sensitive to persistent adverse cognitive effects caused by developmental exposure to the pesticide chlorpyrifos (Levin et al. 2004), much in the same way as rats. Their work, like the present study, demonstrated that zebrafish can replicate a fetal exposure manifesting into adult disease. The well-recognized attributes of zebrafish for developmental studies afford new opportunties to investigate the role of DA and other environmental toxicants that may contribute to a fetal basis for adult disease.
